# Supplementary material for: Early Administration of Bamlanivimab in Combination with Etesevimab Increases the Benefits of COVID-19 Treatment: Real-World Experience from the Liguria Region
Source: J Clin Med. 2021 Oct 13;10(20):4682. doi: 10.3390/jcm10204682 (PMC8538905; doi:10.3390/jcm10204682)
Supplement: Supplementary file 1 [file jcm-10-04682-s001.zip › jcm-1331859-supplementary.pdf]

SUPPLEMENTARY MATERIAL

Table S1. Description of patients who died after BEC infusion.

**Age (years)/ sex**	**Underling conditions**	**Reason for hospital admission**	**Time elapsed between COVID-19 diagnosis and BEC infusion**	**Development of severe/critical illness**	**Period of completed recovery between Covid-19 and death**	**Ultimate cause of death.**
89/M	NYHA class III heart failure; prostatic cancer; Alzheimer's disease	Acute heart failure with pulmonary edema.	1 day	No	No	Acute kidney injury
89/M	Dementia associated with Parkinson's disease; COPD	Left hip fracture due to accidental fall.	1 day	Yes	No	Covid-19 related death
87/F	Stage IV Hodgkin lymphoma receiving palliative care; Hypertension	Rectorrhagia due to thrombocytopenia.	1 day	No	No	Advanced hematological disease
85/F	Heart failure; acute ischemic stroke	New episode of high heart rate atrial fibrillation associated with acute heart failure.	1 day	No	No	Refractory heart failure
83/M	Gastric cancer with peritoneal carcinomatosis, chemotherapy; Cerebrovascular disease; Dementia	Abdominal pain	0	No	Yes (death occurred 30 days after Covid-19 diagnosis)	Advanced gastric cancer
